# Predicting Clinical Dishonesty Among Nursing Students: The Impact of Personal and Contextual Factors

**DOI:** 10.3390/healthcare12242580

**Published:** 2024-12-22

**Authors:** Renata Apatić, Boštjan Žvanut, Nina Brkić-Jovanović, Marija Kadović, Vedran Đido, Robert Lovrić

**Affiliations:** 1 Nursing Institute “Professor Radivoje Radić”, Faculty of Dental Medicine and Health Osijek, Josip Juraj Strossmayer University of Osijek, 31000 Osijek, Croatia; rapatic@mefos.hr; 2 Faculty of Medicine Osijek, Josip Juraj Strossmayer University of Osijek, 31000 Osijek, Croatia; 3 Faculty of Health Sciences, University of Primorska, 6310 Izola-Isola, Slovenia; bostjan.zvanut@fvz.upr.si; 4 Department of Psychology, Faculty of Medicine, University of Novi Sad, 21000 Novi Sad, Serbia; nina.brkic-jovanovic@mf.uns.ac.rs; 5 School of Medicine, The University of Zagreb, 10000 Zagreb, Croatia; marija.kadovic@mefos.hr; 6 Faculty of Health Studies, University of Sarajevo, 71000 Sarajevo, Bosnia and Herzegovina; vedran.djido@fzs.unsa.ba

**Keywords:** academic dishonesty, clinical dishonesty, clinical settings, dishonest behavior, nursing students

## Abstract

Background/Objectives: Numerous studies have examined nursing students’ academic dishonesty; however, there is still a gap in understanding the predictors of such behavior. This study aimed to identify personal (intrapersonal and interpersonal) and contextual factors predicting nursing students’ dishonesty during clinical training. Methods: A two-phase, prospective, predictive study was conducted at a nursing faculty in Croatia. The validated “Mentor Support Evaluation Questionnaire” was used in the first phase to assess students’ evaluations of the quality of mentor support during clinical training. The validated instruments “Optimism/Pessimism Scale” and “Nursing Student Perceptions of Dishonesty Scale” were used in the second phase to examine self-reported dishonesty and its contributing factors. The second phase also investigated the students’ knowledge of the university’s ethical and disciplinary regulations. Results: Of 398 participants, 195 (48.9%) reported engaging in clinical dishonesty. Hierarchical regression analysis identified critical predictors of frequent clinical dishonesty: lack of fear of consequences (*β* = −0.072), positive attitudes toward dishonesty (*β*= −0.081), higher incidence of academic dishonesty in the classroom (*β* = 0.221), lack of knowledge of the university’s regulations (*β* = −0.349), and low quality of mentor support (*β* = −0.430). The final model explained 60.7% of the variance in participants’ clinical dishonesty (*p* < 0.001). Conclusions: The identified predictors indicate that interpersonal factors, i.e., the quality of mentor support, influence students’ clinical dishonesty more than intrapersonal factors (e.g., attitudes or knowledge). Contextual factors (healthcare employment and study overload) were unrelated to clinical dishonesty. This finding can help develop strategies to reduce nursing students’ dishonesty and improve patient safety.

## 1. Introduction

The current competitive academic environment and increasing societal expectations place immense pressure on students to achieve high grades for academic and professional success [1]. This pressure often leads to engaging in academic dishonesty [1,2]. Academic dishonesty involves intentional deception related to one’s work or the work of others [3], representing a failure to adhere to ethical standards, rules, or regulations [4]. This phenomenon in higher education is widespread globally [5,6,7], and nursing students are no exception [2,3,8].

Nursing students’ academic dishonesty has been a subject of research for many decades [6,8,9]. A recent study reported that 91.3% of nursing student participants acted dishonestly twice or more in the classroom, while 32.5% had done so in a clinical setting [2]. Several researchers [2,7,10] have confirmed the correlation between dishonest actions in the classroom (hereafter referred to as classroom dishonesty) and dishonest actions in the clinical setting (hereafter referred to as clinical dishonesty). Therefore, with increases in classroom and clinical dishonesty, healthcare quality and patient safety can be directly and seriously jeopardized [2,8].

Understanding motives for clinical dishonesty among nursing students is critical to the quality of clinical education. Therefore, researchers and academic institutions have long been interested in this subject. Theories, such as Bandura’s social learning theory [11] and Sykes and Matza’s neutralization theory [12], try to explain dishonest behavior. This study is based on the rational choice theory [2], according to which nursing students make decisions by weighing benefits and consequences. Such decision-making is unsafe due to unpredictable circumstances in clinical settings [2,13].

Various personal and contextual factors contribute to nursing students’ clinical dishonesty, including male sex [14], younger age [15,16,17,18], lower grade point averages [14], senior year students [19,20], poor self-image, lack of responsibility, overwork, pressure to succeed, peer competition, stress, fear of failure, lack of awareness, insufficient support from family and friends, unclear definitions of what constitutes dishonest behaviors, the severity of penalties for cheating, inappropriate nurse culture in a clinical setting, poor role models, experiencing ineffective mentoring, etc. [8,15,21,22,23,24,25]. Brown et al. [26] emphasize the importance of the quality of mentor support in encouraging honest and ethical behavior in students, while effective mentor–student relationships can positively influence students’ willingness to report dishonest actions observed in clinical settings. Yadav et al. [27] pointed out that the lack of awareness of professional ethical standards and insufficient ethical training are significant predictors of dishonest behavior among students. Moreover, Park et al. [19] identified that the incidence of clinical dishonesty was related to the number of semesters of clinical practice completed, the type of nursing program, prior knowledge of academic integrity, attitudes toward academic dishonesty, and previous dishonesty on assignments and exams.

Numerous studies have examined dishonesty among nursing students; however, most studies have concentrated on classroom dishonesty more than clinical dishonesty. While several studies have explored clinical dishonesty among nursing students, the factors contributing to such behavior remain insufficiently studied. Identifying these factors is crucial for developing targeted interventions that promote ethical conduct in nursing education while enhancing the profession’s integrity and improving the quality of patient care.

Therefore, this study seeks to fill this gap by identifying predictors of clinical dishonesty. This study examined the relationship between personal (intrapersonal and interpersonal) and contextual (external) factors and the incidence of dishonest actions among nursing students during clinical training. Additionally, hierarchical regression analysis assessed the predictive value of these factors in explaining clinical dishonesty. Intrapersonal factors included participants’ age, gender, level of study, grade point average, religiosity, attitudes toward academic dishonesty, dishonest actions in the classroom, knowledge of the university’s ethical and disciplinary regulations, explanatory style (optimism or pessimism), and self-assessed fear of consequences and punishments. Interpersonal factors involved students’ evaluations of the quality of mentor support during clinical training and their assessments of the level of family support during their studies. Healthcare employment and self-assessed study overload were considered contextual factors.

## 2. Materials and Methods

### 2.1. Study Design

This prospective, predictive study was conducted in two phases at a nursing faculty in Croatia (EU).

The first phase examined participants’ evaluations of mentor support quality during clinical training. According to EU Directive 2005/36/EC [28], clinical training implies the formal education of students in clinical settings under the supervision of clinical mentors.

The second phase examined participants’ self-reported dishonest actions in clinical settings during the study’s first phase and various potential personal and contextual predictors of such behaviors.

This study is part of a five-year (2021–2025) institutional program aimed at the formative evaluation of teaching quality and academic integrity. The program encompasses annual studies and the development of strategies to mitigate dishonesty among nursing students.

### 2.2. Participants

This study included 398 nursing students from the examined higher education institution, of whom 250 (62.8%) were undergraduate students and 148 (37.2%) were graduate students. There were 318 (79.9%) female and 80 (20.1%) male participants. The median age was 23, with an interquartile range of 21–33 years. A total of 213 (53.5%) students were employed in the healthcare sector.

A non-probabilistic purposive sampling method was used, adhering to the inclusion criteria: (a) the participants were undergraduate or graduate nursing students at the examined institution, and (b) the participants had regularly attended clinical training during the study period. This purposive (homogeneous) sampling technique ensured the inclusion of participants directly relevant to the study aims and with the required inclusion characteristics [29]. Therefore, the focus was on nursing students with clinical experience to ensure the investigation of dishonesty in clinical settings. The students completed their clinical training in a hospital or health center, providing direct nursing care to patients under the supervision of mentors (masters of nursing employed at clinics and faculty assistants).

Sample size estimation was conducted using Creative Research Systems’ Sample Size Calculator based on the total number of enrolled nursing students [30]. We aimed for a 3% margin of error, a 95% confidence level, and an alpha of 0.05. The calculation indicated a minimum sample size of 317 participants. Additionally, we confirmed that this sample size would yield a statistical power level of 0.80 for our analyses, including a hierarchical regression with five predictors, which achieved a power level of 0.95 [31]. In total, 398 students participated, resulting in a response rate of 88.4%.

### 2.3. Instruments

The validated Croatian instrument, the “Mentor Support Evaluation Questionnaire” (MSEQ) [32], was used in the first phase of this study. This anonymous questionnaire comprises 25 statements representing desirable mentor competencies and behaviors, such as demonstrating strong knowledge in their field, being available according to student needs, providing feedback on work progress, and showing patience in interactions. Responses are measured on a Likert scale ranging from 1 (strongly disagree) to 5 (strongly agree). Higher scores reflect a stronger agreement with the statements, indicating a higher quality of mentor support. For this study, mentor support quality, as one of the predictor variables, was represented by the overall weighted mean of participants’ responses to the MSEQ, as in the original instrument [32]. The final scores were interpreted using the following pedagogical evaluation criteria: 0–1.9 = very low quality, 2.0–2.4 = low quality, 2.5–3.9 = satisfactory quality, 4.0–4.4 = high quality, and 4.5–5.0 = very high quality. The reliability of the MSEQ, measured by Cronbach’s alpha in this sample, was 0.891.

An anonymous four-section questionnaire was used in the second phase of this study. The first section examined participants’ specific characteristics, including sex, age, year of study, last semester’s grade point average, study workload, healthcare employment, religiosity, and self-assessment of fear of consequences for dishonest actions. The second section of the questionnaire included the validated Croatian “Optimism/Pessimism Scale” (OP Scale) [33] for assessing participants’ explanatory style, which consists of two separate scales: an optimism scale with six items (maximum score: 30) and a pessimism scale with eight items (maximum score: 40). Responses were measured on a 5-point Likert scale (0 = does not apply to me at all; 5 = applies to me completely). Based on the higher percentage between the two scales [33], participants were assigned as either optimists or pessimists. The reliability (Cronbach’s alpha) of the optimism scale in this sample was 0.781, while the pessimism scale had a reliability of 0.820. The third questionnaire section included the validated Croatian version of the “Nursing Student Perceptions of Dishonesty Scale” (CRO-NSPDS) [34], developed by McClung and Schneider [35]. Internal consistency by subscale and overall reliability (Cronbach’s alpha > 0.7) and test-retest reliability (*p* < 0.001) demonstrated the instrument’s high reliability [34]. The CRO-NSPDS comprised 56 items related to dishonest actions, grouped into nine subscales (six classroom subscales and three clinical subscales) (see Table 1).

The incidence of dishonest actions during the examined period was self-reported using a 3-point scale (0 = never, 1 = once, 2 = twice or more), while attitudes toward dishonest actions were self-reported on a 4-point scale (1 = not dishonest, 2 = trivially dishonest, 3 = dishonest, 4 = seriously dishonest) [34]. The classroom items and subscales for determining the incidence of classroom dishonesty and students’ attitudes toward academic dishonesty were used as predictors for explaining the incidence of clinical dishonesty in this study. The reliability (Cronbach’s alpha) of the CRO-NSPDS in this sample was 0.911, with the following subscale reliabilities: Cheating: 0.873, Assistance: 0.780, Cutting Corners: 0.779, Not My Problem: 0.797, Sabotage: 0.889, Test File: 0.801, Perjury: 0.902, Non-Compliance: 0.881, and Stealing: 0.718. The fourth section of the questionnaire contained a written knowledge test on the university’s Ethical Code and the Student Disciplinary Regulations (hereafter referred to as the university’s regulations). The test included ten multiple-choice questions, each with five answer options, of which only one was correct. The overall knowledge level was based on the percentage of correct answers: <49% = unsatisfactory, 50–59% = less than satisfactory, 60–79% = satisfactory, 80–89% = good, and 90–100% = excellent.

### 2.4. Data Collection

**Figure 1:**
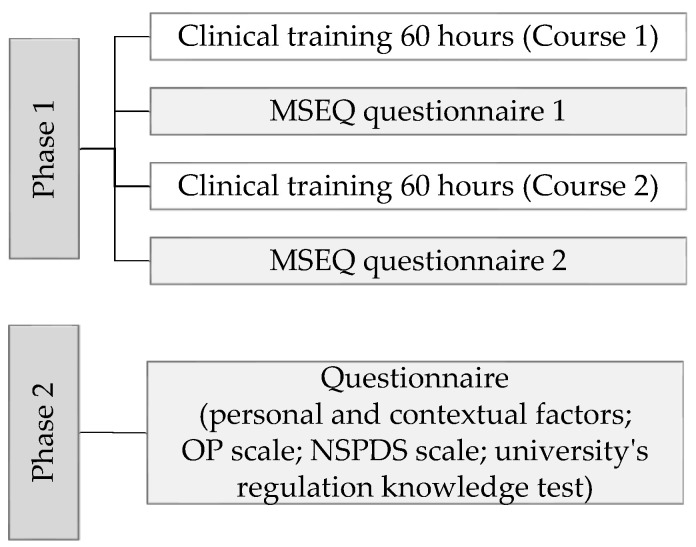
Data Collection Schedule.

In the first phase of this study, participants completed the MSEQ questionnaire twice, a day after the 60 h of clinical training for each course (see Figure 1). The criteria for course selection included: (a) the course must be in the field of nursing, (b) the course consists of 6 ECTS credits or more, and (c) the course must comprise a minimum of 60 academic hours of clinical training. Each participant evaluated the quality of mentor support during 120 academic hours of clinical training. Participation in the questionnaire was voluntary, and participants used a pen-and-paper method to complete it. Once they finished, they placed their completed questionnaires into a locked box before exiting the classroom, after which the researchers took the box. Participants needed an average of 15 min to complete the questionnaire. This study’s first phase involved 402 participants.

All participants who met the inclusion criteria—those who had participated in the first phase—were invited to complete the four-section questionnaire within a week after the end of the first phase (see Figure 1). Participation in the study’s second phase was voluntary, and the data collection methodology and anonymity protocols were followed as in the first phase. Participants needed an average of 45 min to complete the questionnaire. In total, 398 participants completed the questionnaire and written test, providing the final sample for this study. The exact time frames of clinical training and data collection in the first and second phases depended on the curricula of the selected courses.

### 2.5. Data Analysis

Nominal variables were represented using proportions and percentages, while continuous variables were analyzed by calculating the means (M) and standard deviations (SD). Due to significant deviations from normality, as confirmed by the Shapiro–Wilk test, we used the Wilcoxon signed-rank test to evaluate differences between paired dependent variables. The Mann–Whitney U test was used to compare two independent groups, and the Kruskal–Wallis test assessed differences among three or more independent groups. The Spearman correlation coefficient (*rs*) was applied to assess the association between variables. Preliminary tests for the regression model matrix included evaluations of normality, linearity, multicollinearity, homoscedasticity, the independence of residuals, and the identification of outliers. A hierarchical multiple regression analysis was conducted using five-factor models to identify predictors of student dishonesty in a clinical setting. The five-factor model was based on two main criteria: theoretical coherence and empirical statistical robustness. Theoretically, the model reflects established predictors of student dishonesty, spanning personal, academic, social, and contextual factors. Empirically, as a part of the broader preliminary tests, correlation matrices were analyzed to ensure the distinctiveness of variables. Predictors were included hierarchically based on their theoretical relevance and empirical proximity to the outcome variable, allowing for an incremental assessment of each factor’s explanatory contribution. This approach ensures that each predictor adds unique explanatory value to the model, thereby preserving its theoretical coherence and statistical robustness. All *p*-values were calculated using a two-sided approach, with a significance level set at *α* = 0.05. Data analysis was conducted using SPSS for Windows (version 22.0, IBM SPSS, Armonk, NY, USA), the Sample Size Calculator [30], and G*Power software (version 3.1.7, Franz Faul, University of Kiel, Germany) [31].

### 2.6. Ethical Considerations

This study obtained ethical approval from the academic institutions’ Ethics Committees (IRB approval numbers: 2158/97-97-10-23-11 and 2158-61-46-24-174) and was conducted in accordance with the Helsinki Declaration of Human Rights. Participation in this study was voluntary, and participants could withdraw at any time without facing any consequences or penalties. Moreover, this study presented no physical, academic, or emotional risks to the participants. Motivation for participants to participate in the study was provided through a faculty program focused on evaluating the quality of teaching and academic integrity. Participants were exposed to academic dishonesty subjects during several ethics and healthcare courses, which likely further sensitized and motivated their participation in this study. Furthermore, participants in this study had the opportunity to participate free of charge in the faculty’s professional and scientific conferences. Participants completed the scales and questionnaires without the presence of teachers, mentors, or researchers to enhance the objectivity and quality of participants’ responses while minimizing potential feelings of shame and fear. Before completing the questionnaire, participants were informed about the Ethics Committee’s approval and this study’s objectives, procedures, and confidentiality measures, including anonymity and ethical considerations. Each questionnaire included a section for informed consent. Participants’ anonymity was fully protected throughout this study—before, during, and after. Each participant used a unique six-digit code comprising numbers and letters throughout this study. Scales and instruments were validated and used with the necessary permissions, adhering to ethical guidelines to ensure the integrity of the research and the protection of participants.

## 3. Results

### 3.1. Association Between Students’ Attitudes Toward Academic Dishonesty, Personal and Contextual Factors, and the Incidence of Clinical Dishonesty

Of the 398 participants, 375 (94.2%) reported having committed a dishonest act at least once in the classroom, while 195 (48.9%) did so in a clinical setting during the examined period. Participants perceived the “Non-Compliance” clinical subscale as the most dishonest (M = 3.77; range: 1–4), whereas the actions associated with this subscale were the least frequent (M = 0.08; range: 0–2). In contrast, the “Stealing” subscale was considered the least dishonest (M = 3.46), while actions associated with this subscale were the most frequent (M = 0.21) (see Table 2). Furthermore, participants who perceived dishonest actions as trivial practiced these actions significantly more often than those who considered the same actions as seriously dishonest (*p* < 0.001).

The Wilcoxon signed-rank test indicates that participants were significantly more dishonest in the classroom (M = 0.54) than in clinical settings (M = 0.12) (*Z* = −18.03; *p* < 0.001). A Kruskal–Wallis H test indicated that participants with a lower fear of consequences and punishment engaged in clinical dishonesty significantly more often than those with a higher fear (*p* < 0.010) (see Table 3). Furthermore, participants who lacked knowledge of the university’s regulations acted significantly more dishonestly in clinical settings than their more informed peers (*p* < 0.001). Participants who evaluated the quality of mentor support as low engaged in clinical dishonesty significantly more often than those who evaluated it as high (*p* < 0.001). The Mann–Whitney U test indicated no significant difference in the incidence of clinical dishonesty based on participants’ sex (*Z* = −0.387; *p* = 0.699).

### 3.2. Predictors of Students’ Dishonest Actions in Clinical Settings

The correlation matrix showed a significantly weak positive correlation between the incidence of clinical dishonesty and classroom dishonesty (*rs* = 0.403; *N* = 398; *p <* 0.001), suggesting that students prone to classroom dishonesty were more likely to engage in clinical dishonesty. Furthermore, the incidence of clinical dishonesty showed a significant negative correlation with students’ attitudes toward academic dishonesty (*rs* = −0.233; *N* = 398; *p <* 0.001), fear (*rs* = −0.131; *N* = 398; *p <* 0.010), knowledge of the university’s regulations (*rs* = −0.393; *N* = 398; *p <* 0.001), and the evaluated mentor support quality (*rs* = −0.457; *N* = 398*; p <* 0.001). The results indicate that students’ lack of fear of consequences, positive attitudes toward academic dishonesty, high incidence of dishonest behaviors in the classroom, lack of knowledge of the university’s regulations, and perception of mentor support quality as low predict a higher incidence of clinical student dishonesty. No significant correlations were found between clinical dishonesty and age, sex, study program, healthcare employment, grade point average, study overload, family support level, religiosity, or participants’ explanatory style. A hierarchical regression analysis was conducted using five models to examine the predictive value of factors that significantly correlated with the incidence of clinical dishonesty (see Table 4).

All five models were significant (*p* < 0.010), and the percentage of variance explained increased with each subsequent model. The predictor fear of consequences for dishonest actions was in the first model (F(1, 397) = 7.423, R = 0.131, *p* = 0.007). In the second model, the variable reflecting attitudes toward academic dishonesty was added (F(2, 396) = 51.654, R = 0.352, *p* < 0.001). The incidence of classroom dishonesty was included in the third model (F(3, 395) = 57.598, R = 0.478, *p* < 0.001). In the fourth model, knowledge of the university’s regulations was included as a predictor (F(4, 394) = 175.876, R = 0.675, *p* < 0.001). In the fifth and final model, the evaluation of the mentor support quality was added (F(5, 393) = 170.608, R = 0.779, *p* < 0.001).

All predictors remained significant throughout the analysis. The fifth model showed a significant increase in the explained variance, reaching 60.7%, due to the inclusion of the variable that assesses students’ evaluations of mentor support quality. All predictors used in previous models (fear of consequences for dishonest actions, attitudes toward academic dishonesty, and knowledge of the university’s regulations) remained significant and were negatively correlated, while the incidence of classroom dishonesty showed a significant positive correlation. The predictor of students’ evaluations of mentor support quality had a significant effect on the criterion (*β* = −0.430, *p* < 0.001), showing a negative correlation (see Table 4).

These results suggest that a lack of fear of consequences, positive attitudes toward academic dishonesty, a high incidence of dishonesty in the classroom, a lack of knowledge of the university’s regulations, and perceived low-quality mentor support predict a higher incidence of clinical dishonesty among nursing students.

## 4. Discussion

This study aimed to identify personal and contextual predictors contributing to a higher incidence of clinical dishonesty among nursing students. The results reveal that 48.9% of participants acted dishonestly at least once in clinical settings during the examined period. The result is slightly lower than the results in Australian (50%) [16], American (54%) [36], Korean (65.8%) [19], and Iranian (89.1%) [8] studies. Additionally, the mean incidence of dishonest actions across subscales and the overall NSPDS score (0.12) may appear low given the scale’s range from 0 to 2, but these results are rather concerning. Clinical settings represent high-risk environments where dishonest actions can directly jeopardize patient safety and compromise healthcare quality [2]. Research on nursing students’ clinical dishonesty remains a global priority, and identifying universal causes for such behaviors seems unachievable [2]. Previous studies have reported significant associations between dishonest actions and factors such as sex, age, year of study, and grade point average [2,8,14,15,16,18]. However, the present study found no significant relationship between clinical dishonesty and personal factors such as age, sex, study program, healthcare employment, grade point average, religiosity, level of family support, and explanatory style, confirming other studies [19,36,37,38,39]. Although study overload was not associated with clinical dishonesty in this study, the impact of student overload may vary depending on the type and difficulty of the tasks [2,40]. Study overload can be reduced by optimizing tasks, e.g., reducing redundant theoretical tasks and introducing more activities that integrate theory with practice. A better balance between theoretical and practical tasks can reduce stress, increase student engagement, and prevent potential ethical dilemmas [40]. Future research should consider how different aspects of overload and institutional climate affect nursing students’ behavior in clinical settings. This study identified five key predictors that significantly contribute to the high incidence of clinical dishonesty among participants. A total of 60.7% of the criterion for dishonest actions was explained. The details of these predictors are described further in the text.

The strongest predictor of the incidence of clinical dishonesty among participants was the evaluated mentor support quality. In this study, 8.1% of participants evaluated the mentor support quality as low or very low, while 17.3% found the quality satisfactory. Mentor support is crucial at every stage of nursing education, as it influences students’ knowledge, skills, attitudes, and professional development while fostering a culture of academic integrity [41,42,43]. The results of this study indicate that mentor support significantly reduced the impact of other predictors, especially students’ attitudes toward academic dishonesty. This result indicates a strong influence of mentor support on students’ attitudes and the probability of students’ dishonest actions in the clinic. According to Bandura’s social learning theory, nursing students adopt behaviors they have been exposed to in clinical settings [11,44]. Besides knowledge and skills, the quality of mentor support encompasses modeling ethical behavior and creating an environment for students to internalize these behaviors as their own [45]. This mentor competency is essential in clinical settings where students often face complex ethical dilemmas and can seek guidance from mentors [32]. However, students sometimes may be exposed to unprofessional, substandard, or absent role models [7,46]. If mentors do not offer a clear and consistent model of ethical behavior, students can become more prone to dishonest actions. Potential problems arising from insufficient mentor support can be analyzed through Ross’s and Mitnick’s agency theory [47]. The first problem is information asymmetry, exacerbated by mentors’ inadequate presence and engagement. This can lead to insecurity and potentially irresponsible and dishonest behavior among students. Another problem is the motivation to perform procedures following ethical standards, which may be lacking due to the absence of constructive feedback and mentor support. The third issue is the absence of control; with insufficient mentor support, control mechanisms become less effective, increasing the likelihood of dishonest actions. Finally, students may feel less responsible and not perceive the consequences of their actions as there is no adequate supervision [44]. All these issues jeopardize clinical education and healthcare quality [7,42,48]. The evaluated mentor support quality predictor highlights the need to organize educational and motivational activities for mentors’ quality improvement while ensuring sufficient mentors for students’ support during clinical training. Productive mentoring training needs to be integrated into the curriculum and training for mentors [2]. Introducing structured feedback mechanisms and ongoing mentor training could help address potential gaps in mentor support. Furthermore, fostering an institutional culture that prioritizes mentoring and ethical standards can lead to a more supportive environment and reduce the possibility of clinical dishonesty [49].

It is important to emphasize that besides mentor support, other interpersonal factors can also influence the shaping of student behavior during clinical training, e.g., strong peer influence on students’ propensity for dishonest actions, especially in stressful clinical settings where social pressure shapes students’ decisions [50]. Since student behavior reflects the institutional climate, influence from mentors and institutions promoting open communication, accountability, and defined ethical norms may reduce the incidence of dishonest actions by students [51].

The second predictor was students’ knowledge of the university’s ethical and disciplinary regulations. In this study, more than half of the participants (56.1%) showed unsatisfactory or less than satisfactory understanding of these regulations on the knowledge test, while 43.9% demonstrated satisfactory to excellent knowledge. Besides the high percentage of participants with a lack of knowledge, these results are particularly concerning because students’ performance on the test may not genuinely reflect a profound grasp of the concepts related to academic dishonesty [52]. Some authors [3,6] have highlighted nursing students’ insufficient understanding of the scope of academic dishonesty and its associated policies and penalties, particularly when acting dishonestly according to university standards. Integrating experiential learning methods, such as simulations and case-based discussions, has improved students’ ability to face ethical dilemmas in practice and develop critical thinking skills [53]. Moreover, students with a greater understanding of institutional policies are significantly less likely to act dishonestly [54]. Honor codes and academic environments characterized by integrity have been demonstrated to be critical for effectively addressing academic dishonesty. At the same time, familiarity with the university’s regulations has been shown to mitigate such behaviors [6,21,36,54]. Therefore, these materials should be introduced early in students’ studies through formal and informal courses, meetings, or workshops to improve student learning. The main goal of these initiatives is to encourage understanding, analysis, and critical reflection rather than merely repeating information.

The third predictor that significantly contributes to clinical dishonesty is the incidence of dishonest behavior in the classroom. Although such behaviors may initially appear harmless, the results of this study suggest that they can serve as a potential “first step” toward clinical dishonesty among nursing students. Acting dishonestly in the classroom can result in knowledge gaps and reasoning deficits, which may jeopardize patient safety [2]. In this study, 94.2% of participants reported engaging in dishonest classroom behaviors at least once during the examined period. This result is consistent with findings from prior studies [2,8,12,15,19]. Compared to clinical settings, the lower incidence of dishonest behavior among participants in the classroom aligns with expectations. According to the rational choice theory, students determine whether to act dishonestly by weighing the potential consequences, which are significantly more severe in clinical environments [2,8,35]. Krueger [36] asserts that nursing students who perceive dishonest behavior in the classroom as highly unethical are also likely to consider clinical dishonesty as being the same. Additionally, some studies indicate that students acting dishonestly in the classroom are likely to show similar tendencies in clinical settings [2,10,19,36]. Some authors have established a connection between the lack of knowledge acquired from dishonest classroom behaviors and subsequent clinical dishonesty and incompetence [8,12]. Students who practice dishonest behavior in the classroom are more likely to engage in unethical manipulations in the clinical setting during their future professional work [17]. Therefore, initiatives to reduce clinical dishonesty and improve patient safety should be integrated into nursing curricula on time, starting with early preventive measures in the classroom and extending through clinical education rather than being restricted only to clinical settings [55].

The fourth predictor identified in this study was a fear of consequences and punishments for dishonest actions. More than half (52%) of participants indicated no or moderate fear of consequences when acting dishonestly. According to Bandura’s social learning theory, early education about the penalties for dishonesty enhances students’ observational learning [11]. Students who understand the consequences of dishonest actions are more likely to avoid such actions. A significant correlation exists between a low incidence of dishonesty and faculty discussions regarding honor codes and violation penalties [11]. Several authors [8,11,16] highlight that the fear of being caught and the imposition of severe penalties are significant deterrents to dishonest actions. In a study conducted by Birks et al. [16], approximately 90% of nursing students indicated that severe penalties would discourage dishonest actions. Furthermore, Kiekkas et al. [15] argue that the absence of severe consequences for cheating may foster an environment conducive to student dishonesty. However, it is essential to note that excessive fear of consequences and penalties may compel students to conceal or deny their mistakes in clinical settings [56]. Therefore, higher education and clinical institutions must nurture an environment where students respectfully approach university and clinical regulations rather than fearing the consequences of their violations.

The fifth predictor of clinical dishonesty identified in this study was participants’ attitudes toward academic dishonesty. The results showed that participants acted dishonestly least frequently with regards to the “Noncompliance” subscale, which they perceived as the most dishonest. In contrast, they frequently practiced dishonest actions from the “Stealing” subscale, which they considered the least dishonest. These results are expected, as students’ behavioral patterns are significantly influenced by their perceptions, beliefs, attitudes, and values [2]. According to Sykes and Matza’s theory of neutralization, students employ specific techniques to justify their dishonest actions, rationalizing them as logical and fair to alleviate feelings of guilt [2,3]. Furthermore, students often decide whether to engage in dishonest actions based on their attitudes and after assessing the severity of potential consequences [2,13]. Research suggests that the more unethical a behavior is perceived as being, the less frequently students engage in it [20,57]. In a South Korean study, Lee et al. [20] found a significant correlation between students’ attitudes toward academic dishonesty and their dishonest actions. The more negative students’ awareness and attitudes toward academic dishonesty, the lower their engagement in misconduct [20]. Additionally, a Canadian study confirmed that students’ attitudes, subjective norms, and perceived behavioral control were the strongest predictors of clinical dishonesty [57]. Nursing students with a more robust positive attitude toward academic integrity are likelier to adhere to honesty in their clinical practice. In contrast, those with positive attitudes toward academic dishonesty are more prone to engage in misconduct [57]. It is crucial to note that mentor support quality in this study significantly impacted the participants’ attitudes and likelihood of practicing dishonest actions.

Although a fear of consequences and attitudes toward academic dishonesty individually have a weaker contribution, the overall role of these two predictors in the model remains significant. Additional testing showed that their removal significantly reduced the overall percentage of explained clinical dishonesty. Theoretically, these predictors remain key in the relevant literature as they provide valuable insights into the complex mechanisms that shape academic and clinical dishonesty. Their presence in the model ensures compliance with existing theoretical frameworks and enables a more comprehensive approach to the analysis of the predictors. Practically speaking, their inclusion points to developmental opportunities for targeted interventions that may contribute to reductions in dishonest behavior in the clinical setting, regardless of their small individual impacts. In particular, their integration into ethics education programs or alignment with institutional policies could improve the effectiveness of strategies to reduce dishonest behaviors. Further research into their connection with ethical education and institutional culture could provide more intelligible practical implications.

Following the above, academic institutions should prioritize their students’ cognitive and psychomotor learning outcomes and promote effective learning that shapes students’ attitudes, social values, and ethical principles. The establishment of honor codes is a strategic approach to deterring academic dishonesty and may significantly influence students’ perceptions and attitudes toward such behavior [2]. Experiential teaching methods, such as case-based learning, are highly effective in developing students’ ethical competence by promoting critical thinking, empathy, and ethical decision-making [53]. Promoting ethical sensitivity and awareness of honor codes through dedicated educational modules, courses, and workshops will cultivate a culture of integrity.

### 4.1. Limitations of This Study

This study has several limitations. Firstly, it was conducted at a single university in Croatia; however, it incorporated three nursing programs in different cities and represents one of the most prominent institutions in the country offering nursing education. Secondly, students’ behaviors were self-reported, while direct observations could provide more accurate and comprehensive data. However, observing students with their consent may affect their natural behavior and introduce bias. Additionally, ethical dilemmas might arise if students are unaware they are being monitored. Third, the data were collected using validated questionnaires and scales. Future studies should consider applying mixed research methods, which are more likely to provide deeper insights into the investigated phenomenon. Finally, the mean (SD) was used to represent the incidence of clinical dishonesty despite the narrower range of the scale. However, preliminary analyses confirmed that this approach provided a precise and informative insight into subtle differences between participants, which was crucial for achieving the study aims.

### 4.2. Contribution of Study Results to Education and Clinical Practice

This study could positively affect nursing education and everyday clinical practice. Identifying factors contributing to clinical dishonesty highlights the need for specific changes in educational programs to promote and strengthen ethical values.

The results emphasize the need for enhanced training and support for clinical mentors, who are crucial in shaping students’ ethical values. Mentors serve as professional role models, influencing students’ attitudes and behaviors. Therefore, higher education institutions should prioritize investing in their mentors’ professional, ethical, and moral development through supportive and educational initiatives.

It is crucial to integrate structured feedback mechanisms and mentor training into the curriculum, focusing on specific ethical dilemmas and the mentor’s role in solving them. In addition, it is essential to implement a system based on student feedback, which will enable mentors to obtain a more realistic picture of the support they provide, identify areas for improvement, and adapt methods to reduce clinical dishonesty and shape students’ ethical values. Academic and clinical institutions should continuously support mentors through regular education and counseling. Academic institutions can organize specialized workshops and seminars on ethics, while clinical institutions can provide mentors with ongoing supervision and access to resources that will help them develop professional skills. These measures can significantly contribute to strengthening the ethical dimension of mentoring and reducing clinical dishonesty among students.

The students’ lack of knowledge of the university’s regulations, one of the identified significant predictors, indicates the importance of early implementation of ethical content in nursing curricula. These approaches can better prepare students to confront and resolve ethical dilemmas in clinical practice, ultimately improving healthcare quality with a focus on patient safety. Student dishonesty in the classroom, as identified in this study, is one predictor of increased clinical dishonesty.

This suggests a critical need for early education interventions to familiarize students with ethical norms, regulations, and disciplinary procedures related to academic dishonesty. Educational interventions must focus not only on the transfer of knowledge but also on the development of ethical decision-making skills that will be crucial for students in complex clinical settings. The inclusion of experiential learning tools, such as case-based learning or ethics simulations, would allow students to actively engage in realistic scenarios and practice ethical decision-making before facing such challenges in clinical settings. The introduction of an honor code and the implementation of ethics workshops aimed at developing ethical sensitivity among students represent key steps toward this goal. Higher education institutions for nursing should consider these predictors to effectively prepare students for professional roles that respect ethical principles and ensure safe, high-quality healthcare.

## 5. Conclusions

This study revealed that almost half of the participants acted dishonestly in clinical settings at least once during the examined period. The incidence of clinical dishonesty was not associated with examined contextual factors nor with half of the personal factors. However, five predictors of student clinical dishonesty incidence were identified: evaluated mentor support quality as low, lack of fear of consequences for dishonest actions, positive attitudes toward academic dishonesty, more frequent dishonest behavior in the classroom, and lack of knowledge of the university’s ethical and disciplinary regulations.

These results highlight the importance of promptly incorporating ethical content into nursing curricula and involving mentors in these educational activities. This approach ensures that students receive high-quality mentor support during their clinical training. Additionally, it is essential to continually educate students about ethical standards and appropriate disciplinary actions related to academic dishonesty. Implementing these measures is vital for preparing students for professional roles that demand adherence to moral principles and for ensuring safe, high-quality healthcare.

## Figures and Tables

**Table 1:** Descriptions of classroom and clinical CRO-NSPDS subscales.

CRO-NSPDS	No. of Items	Description
Classroom subscales		
1. Cheating	12	Attempts to succeed on tests or assignments without completing the work.
2. Assistance	5	Improving one’s performance by collaborating with others.
3. Cutting Corners	5	Taking steps to minimize the amount of work required.
4. Not My Problem	4	Recognizing academic dishonesty in others but failing to report it.
5. Sabotage	4	Hurting another person’s academic work.
6. Test File	2	Keeping or using previous exams or collections of test questions.
Clinical subscales		
7. Perjury	13	Creating or presenting false information to mislead others.
8. Non-compliance	8	Ignoring established guidelines, rules, or expectations.
9. Stealing	3	Taking something without proper authorization or ownership.

**Table 2:** Incidence of clinical dishonesty concerning student attitudes toward academic dishonesty.

CRO-NSPDS Clinical subscales	Attitudes Range (1–4) *		Clinical Dishonesty Incidence Range (0–2) **	
	Mean	SD	Mean	SD
Perjury	3.65	0.56	0.12	0.33
Non-compliance	3.77	0.34	0.08	0.27
Stealing	3.46	0.37	0.21	0.34
Overall clinic	3.66	0.38	0.12	0.28

* Attitude scale: (1 = not dishonest, 2 = trivially dishonest, 3 = dishonest, 4 = seriously dishonest). ** Incidence scale: (0 = never, 1 = once, 2 = twice or more).

**Table 3:** Association of students’ personal and contextual factors with the incidence of clinical dishonesty (*N* = 398).

Personal and Contextual Factors		*N* (%)	Clinical Dishonesty Incidence (Range 0–2)	*p* Value
			Mean (SD)	
Study	BSc	250 (62.8)	0.04 (0.23)	0.752 ^†^
	MSc	148 (37.2)	0.06 (0.25)	
Healthcare employment	Yes	213 (53.5)	0.29 (0.25)	0.679 ^†^
	No	185 (46.5)	0.28 (0.26)	
Grade point average	2.5–3.4	49 (12.3)	0.41 (0.36)	0.868 ^††^
	3.5–4.4	253 (63.6)	0.32 (0.22)	
	4.5–5.0	96 (24.1)	0.30 (0.26)	
Study overload	Low	1 (0.3)	0.27 (0.26)	0.650 ^††^
	Moderate	268 (67.3)	0.33 (0.25)	
	High	129 (32.4)	0.33 (0.26)	
Level of family support	Low	4 (1.1)	0.35 (0.27)	0.258 ^††^
	Moderate	373 (93.7)	0.32 (0.24)	
	High	21 (5.2)	0.30 (0.22)	
Religiosity	Yes	343 (86.2)	0.33 (0.26)	0.388 ^†^
	No	55 (13.8)	0.31 (0.20)	
Explanatory style (OP scale)	An optimist	291 (73.1)	0.36 (0.22)	0.187 ^†^
	A pessimist	107 (26.9)	0.40 (0.31)	
Self-assessment of fear of consequences for dishonest actions	None	9 (2.3)	0.16 (0.35)	<0.010 ^††^
	Moderate	198 (49.8)	0.08 (0.22)	
	High	191 (47.9)	0.07 (0.16)	
Knowledge of the university’s regulations (knowledge test)	Unsatisfactory *	58 (14.6)	0.67 (0.00)	<0.001 ^††^
	Less than satisfactory *	165 (41.5)	0.65 (0.20)	
	Satisfactory *	127 (31.9)	0.17 (0.16)	
	Good *	32 (8.0)	0.06 (0.04)	
	Excellent *	16 (4.0)	0.02 (0.04)	
Evaluation of the mentor support quality (MSEQ questionnaire)	Very low **	11 (2.8)	0.69 (0.65)	<0.001 ^††^
	Low **	21 (5.3)	0.37 (0.19)	
	Satisfying **	69 (17.3)	0.12 (0.13)	
	High **	141 (35.4)	0.05 (0.06)	
	Very high **	156 (39.2)	0.02 (0.03)	

* Unsatisfactory (<49%); less than satisfactory (50–59%); satisfactory (60–79%); good (80–89%); excellent (90–100%). ** Very low (0–1.9); low (2.0–2.4); satisfactory (2.5–3.9); high (4.0–4.4); very high (4.5–5.0). ^†^ Mann–Whitney Test; ^††^ Kruskal–Wallis Test.

**Table 4:** Hierarchical multiple regression models of the clinical dishonesty prediction.

Models/Predictors	*β*	*t*	R	*R^2^*	∆R^2^	|F
**Model I**			0.131 *	0.017	0.17 *	7.423
Fear of consequences	−0.131 **	−2.724				
**Model II**			0.352 ***	0.124	0.107 ***	51.654
Fear of consequences	−0.118 *	−2.585				
Attitudes toward academic dishonesty	−0.327 ***	−7.187				
**Model III**			0.478 ***	0.228	0.105 ***	57.598
Fear of consequences	−0.113 *	−2.654				
Attitudes toward academic dishonesty	−0.295 ***	−6.873				
Dishonesty in the classroom	0.325 ***	7.589				
**Model IV**			0.675 ***	0.455	0.227 ***	175.876
Fear of consequences	−0.085 *	−2.361				
Attitudes toward academic dishonesty	−0.177 ***	−4.762				
Dishonesty in the classroom	0.258 ***	7.089				
Knowledge of the university’s regulations	−0.498 ***	−13.262				
**Model V**			0.779 ***	0.607	0.142 ***	170.608
Fear of consequences	−0.072 *	−2.332				
Attitudes toward academic dishonesty	−0.081 **	−2.454				
Dishonesty in the classroom	0.221 ***	7.013				
Knowledge of the university’s regulations	−0.349 ***	−10.120				
Mentor support quality	−0.430 ***	−12.191				

*β* = beta regression coefficient; *t* = *t*-test statistic; R = multiple correlation coefficient; *R*^2^ = coefficient of determination; ∆R^2^ = R Square Change; *|*F = F-ratio. * *p* < 0.050; ** *p* < 0.010; *** *p* < 0.001.

## Data Availability

All data generated and analyzed during the current study are available from the corresponding author upon reasonable request.
